# Services in the self: embodied labor and the global bioeconomy

**DOI:** 10.1186/s40504-015-0027-x

**Published:** 2015-09-14

**Authors:** Christian Haddad

**Affiliations:** University of Vienna, Wien, Austria

**Keywords:** Bio-economy, Labor, Post-fordism, Artificial reproductive technologies, Clinical trials, Regenerative medicine, Contract law, Sociology of bioethics

## Abstract

This review article discusses Melinda Cooper and Catherine Waldby’s recent book, *Clinical Labor*. *Tissue donors and research subjects in the global bio-economy* (Duke, 2014), as a topical contribution to the literatures on the bio-economy, and to studies of life sciences, society, and policy more generally. The article contextualizes the book within existing literatures (1) and thoroughly considers its conceptual approach as well as its findings (2). Further, it discusses its value as a contribution, arguing that clinical labor also presents an intriguing framework for further research, thereby suggesting some possible directions (3).

## Book details

Cooper, Melinda, and Catherine Waldby. *Clinical labor: Tissue donors and research subjects in the global bioeconomy*. Duke University Press, 2014. (296 pages, ISBN: 978–0822356226).

## Bio-economic aspirations and clinical labor

Roughly the last decade has seen the growing interest in a new object of social science inquiry and socio-political imaginary, the “bio-economy”. As imaginary, the bio-economy is perhaps best captured by the OECD’s aspiration to “turn the disruptive potential of biotechnology to economic advantage“ (OECD 2009: 7). The bioeconomy has become a matter of topical concern and a target of various policy programs, strategy papers and public-private initiatives. Framed as a solution to a series of crises linked to problems of poor productivity, declining profits, and an ever-growing appetite for innovation, the bioeconomy has come to signify a crucial transformation in today’s knowledge-based economies (Scharper-Rinkel 2012). Policy makers and stakeholders puzzle about how the life sciences are to be successfully commercialized, what kinds of legal and technical infrastructures need to be put in place, and what kinds of specialized labor force needs to be created to bring about a thriving bioeconomy (Hilgartner 2007).

Accordingly, the bioeconomy has also captured the interest of the social sciences. Sociologists, anthropologists and STS scholars, among others, have started to explore the entanglements between life sciences and political economy (see Helmreich 2008 for an overview), centering on questions such as: How is value attached to newly emerging life forms, such as patented microbes or stem cells? How, and to which extent, are markets created for entities hitherto excluded from market relations, such as human tissues and body parts? How do these “tissue economies” (Mitchell and Waldby 2008) play out in globally refracted networks? In which ways do these novel articulations of knowledge and value impact on social relationships, identities, and forms of subjectivity? (Thacker 2005, Sunder Rajan 2006, 2012; Cooper 2008; Birch 2012; Dussauge et al. 2015)

In this body of work, the bio-economy signifies more than simply a new branch or market segment (Lettow 2012), that is to say, an “economy of the life sciences” and its frictionless commercialization of biomedical knowledge and productivity. For instance, some contributions have suggested to explore a “biotechnological mode of production” corresponding with the emergence of a “post-Fordist” political economy (Thompson 2005; Cooper 2008). Post-Fordism typically refers to a post-industrial regime of capitalist relations characterized by the rise of the service sector, increased financialization and a “knowledge-based” type of valorization through the creative industries – but equally by “new modes of biomedical production focused on innovation value and newly defined contractual rights in the body” (Cooper & Waldby 2014: 3).

Other contributions argue that the bioeconomy amounts to a political epistemology: a new mode of ordering socio-technical relations, regimes of truth, and systems of values (Sunder Rajan 2006, 2012; Larsen 2007; Cooper 2008).

Melinda Cooper and Catherine Waldby’s book contributes to this scholarship by zeroing in on the question of labor. In the policy discourses of the bioeconomy, the notion of labor predominantly refers to the cognitive and technical skills of the knowledge worker (researcher, engineer, project coordinator, etc.). This narrow focus silences the role of a broad range of other subjects performing “clinical labor”, thereby significantly contributing to biomedical innovation and bioeconomic generation of value.

Against this background, the authors suggest that despite the abundance of reflections on the bioeconomy in academic and policy discourse, “few have explored the very material ways in which the in vivo biology of human subjects is enrolled into the post-Fordist labor process” (Cooper & Waldby 2014: 7). Zeroing in on central forms and practices of contemporary biomedicine (such as reproductive medicine and the clinical trials industry), Cooper and Waldby thus explore clinical labor as a set of largely unacknowledged productive relations and account for its rise and saliency.

## A cartography of global clinical labor

*Clinical Labor* is divided into three main parts. The first part introduces the analytical perspective in theoretical and historical regard. Conceptually, the term “clinical labor” facilitates a double critique. On the one hand it serves to assemble and articulate various productive activities that tend to be dealt with separately: the participation in clinical research trials, the donation of bodily materials and tissue samples for research or for reproductive purposes, the carrying out of gestational surrogacy, the sharing of valuable bio-information. Research subjects and tissue donors constitute the “clinical laborers” of the global bio-economies, as they all perform “services in the self”: risky, embodied forms of labor critical to the overall bio-economic enterprise. On the other hand, the concept of clinical labor brushes conventional theories of labor, bioethics and innovation against the grain: insofar as these theories ignore or actively exclude such embodied work from the realm of formal labor, they fail to conceptualize and thus come to terms with clinical labor, analytically and theoretically, as a crucial set of social productive forces.

The first chapter establishes “clinical labor” as a novel concept and traces its emergence through a genealogy of tentative articulations of life, labor and value – including Marx’s value theory of labor, Chicago school economics, and Italian post-operaismo. Further, Cooper and Waldby elaborate crucial omissions and silences in the academic as well as political and policy discourses, which, in a way, have circumnavigated the question of clinical labor altogether. These silences prominently include bioethics to the extent that bioethics has sought to protect subjects of biomedicine precisely by shielding them from the spheres of market forces and paid wage-labor (p. 7–8). Chapter 2 shows how the post-Fordist business model has redefined the relationships of labor, risk and social reproduction. Precipitated by various currents, including the rise of human capital theory, processes of de-unionization and heavy recourse to legal contractualism, the independent contractor, “emblematic of precarious” as well as highly risky labor, has emerged as salient figure of post-Fordist economies (p. 27).

In the global bioeconomy, independent contractors are contractors of their ‘biological capital’ […], obliged to assume both the economic and corporeal risks of the biomedical innovation economy.” (p. 27–28). Discriminating between the speculative risks of innovation assumed by scientists and investors and the “visceral risks” of the clinical laborer, the bioeconomy in turn “depends on the devolution of uninsurable, embodied risks to the clinical laborer” (p. 32). Moreover, these embodied risks are distributed unevenly within societies and across the globe, capitalizing on asymmetries and hierarchies within and across categories of class, gender, and ethnicity. Perhaps unsurprisingly, the economic precariat of deindustrialization, women, and ethnic minorities assume the predominant share of the embodied risks of biomedical innovation. This is the case, as subsequent chapters detail, because for those many “freed” from social security and workplace insurance of the declining welfare state, clinical labor provides a necessary source of subsistence, creating income opportunities for the unemployed and working poor populations in their capacity as experimental or reproductive bodies in a global service economy (p. 81).

Defined broadly as the worker’s assumption of biopolitical risks (p.18), clinical labor it is nevertheless a historically specific concept that emerges against the background of post-Fordism. Clearly, individuals and entire groups have been systematically exploited for medical research from the 19th century onwards. Historically, prototypical clinical labor has been tried and tested in secluded spaces institutionally separated from the political economy proper and from formal labor relations (such as the prison, the asylum, or the nuclear family). However, with the disintegration of “Fordist” productive relations – including the denationalization of production, de-unionization of labor and the de-differentiation of spheres of production, reproduction, and consumption – clinical labor has become a ubiquitous form of informal, contractual labor heavily entwined with neoliberal globalization, its social and economic restructuration, and its specific modes of appropriation and exploitation.

Hence, the remainder of the book investigates how the biomedical industries draw on, actively exploit, and (re-)produce the stratified social relations in a global post-Fordist bioeconomy. In doing so it jointly explores the (bio-)technical and legal preconditions that have enabled the rise of clinical labor as a global phenomenon.

### From reproductive to regenerative labor

The second and third part of the book engage empirically with two distinct, though related fields of the life sciences. Part II zeros in on reproductive biology, a sphere that has for long been excluded, ideologically and legally, from the sphere of market relations. Yet, while reproductive labor as typically feminized work is deeply rooted in the convoluted histories of class struggle, colonialism and sexual relations of domination, “today the labor of human reproductive biology has become precisely a form of economic labor in certain key sectors of the bioeconomy.” (p. 33). Chapter 3 explores artificial reproductive technologies (ART) and how their emergence in the US has enabled the disentanglement of certain body parts such as oocytes from the body proper. Blurring the boundaries of social and biological reproduction, ART manages to reorganize certain reproductive processes – such as fertilization or the entire gestation process *qua* surrogacy – as services *in* the self that can be sourced out to "free and independent" contractors. In that, ART has not only technologically altered biological reproduction, but moreover has given rise to family relations beyond the conventional norms of kinship. Yet, these shifts were possible only through the simultaneity of technological advances and the socio-legal and economic realignment of reproduction on a global scale. Chapter 4 explores the ways these novel technologies have spread from the US to various other countries worldwide, including detailed reports on Eastern European countries, Spain, and India. It shows how the uptake and selective organization of ART in respective countries is related to the country’s position in the globally stratified biopolitical economy. A newly emerging “fertility tourism” characterizes global biopolitical relations, and “reproductive outsourcing is profoundly intertwined with the post-Fordist reorganization of other kinds of feminized labors and the rendering of formally domestic, privatized aspects of household reproduction as service labor, itself often transnationalized” (p. 87). Finally, Chapter 5 traces a relocation of feminized bioeconomic tasks from ART and reproductive labor to stem cell technologies and their corresponding form of regenerative labor. As a thriving branch of the bioeconomy, stem cell research largely depends on cells and tissue donated for research, among them IVF embryos, aborted fetuses or cord blood. In most cases, women are conceptualized as voluntary participants and altruistic donors, even though these “donations” involve considerable risks and efforts. Moreover, the material practice of giving regenerative tissue to research simultaneously performs a transaction of yet another kind: typically, the women-donor thereby transfers nonreversible ownership rights to the recipient, be it a team of scientists or a biotech company, thereby receding from all claims including future profits gained through biotechnological manipulation and commercialization of disentangled cells and tissues.

### Experimental labor

Part III explores experimental clinical labor done by human research subjects and patients in pharmaceutical clinical trials. Today’s ever growing drug consumption involves growing markets in clinical trials, and thus the need for more clinical research subjects. While the global search for subjects has become a pressing organizational and ethical problem, clinical trial participation has largely remained to be considered as a voluntary contribution to research and thus excluded from the realm of labor. That notwithstanding, a multitude of subjects are motivated by financial “compensation” or even “undertake clinical trials as a form of paid work or work for health care” (p. 117), particularly in contexts where access to medical checks and basic health care is scarce or simply unavailable. Cooper and Waldby begin by reconstructing the history of human subject research in post-war America. Chapter 6 shows how clinical trials have moved from wartime exceptionalism into the secluded space of the carceral system. Later, as prison-based experiments came under considerable critique, clinical research was moved into private medical centers and contract research organizations. The growth of the clinical trials industries notwithstanding, experimental labor has been kept away from the sphere of official labor relations – and bioethics has been a major force in maintaining this legal and discursive separation.  From the 1990s onwards, newly liberalized countries and emerging economies have provided new experimental spaces for offshoring pharmaceutical production and clinical labor (Chapter 7). In a sense, the crisis of pharmaceutical research was partially resolved by “contracting out” experimental labor (and its embodied experimental risks) both institutionally and nationally.

Finally, the last chapter turns to a set of novel strategies and relations in drug innovation that walk hand in hand with a realignment of the clinic and its central place in the pharmaceutical business model. These strategies consist in opening up the confined clinical-experimental space towards a more distributed form of public experiment. On the one hand, patient groups in the US and elsewhere have increasingly claimed a right to assume experimental risks by accessing investigational drug products. In this effort, some have formed alliances with pharmaceutical companies, or enjoy the support of libertarian think tanks and their deregulatory agendas. On the other hand, novel, mostly web-based strategies have sought to enroll the public in a collective effort of data generation facilitating some sort of open source model of biopharmaceutical innovation. Patients are asked to share and meticulously document their experiences with medication regimes, thus collecting tons of “post-marketing” data valuable to the pharmaceutical industry. Thereby the public is being reconfigured as an “unwaged, highly skilled labor market” (p. 218), further blurring the lines between clinic, market and society.

## Discussion: A diagram of post-Fordist life, value and labor

*Clinical Labor * offers detailed insights of how clinical trials, ART and regenerative medicine unfold aggressively in an expansive global post-Fordist economy. In a way, the argumentative structure of *Clinical Labor* resembles Eduardo Galeano’s famous *Open Veins of Latin America* (Galeano 1998). Galeano’s book details the brutal colonial history of Latin America, by reconstructing, chapter by chapter, each country’s proper history of productive relations through the lenses of distinct commodities such as sugar cane, coffee or silver. While each separate chapter provides a compelling “stand alone” analysis, the true force of Galeano’s narrative unfolds only if these strands are articulated in an overall argument, thus revealing the overarching structures and patterns of political-economic domination that emerge as forceful and concerted system. Similarly, *Clinical Labor* provides a series of differentiated analyses, organized in separate chapters, that each deliver thorough analyses of different technologies (ART, clinical trials, etc.) articulated within particular socio-economic and cultural contexts (the US, China, Eastern Europe, etc.), thereby producing varied subjects (the oocyte vendor, the uninsured clinical research volunteer, etc.) and different forms of clinical labor (reproductive, regenerative, experimental, etc.). This differential perspective in itself is a welcome contribution to the state of knowledge in these thematic fields of scholarship. But akin to *Open Veins*, the full extent of Cooper and Waldby’s argument lies in the theoretical perspective on clinical labor as a general phenomenon and trajectory of the intertwined biopolitical (re-)organization of relationships between biomedicine and the post-Fordist economy. If one abstracts from the contingencies of particular technologies, certain biocapital branches, or regional specificities, “clinical labor” emerges as a powerful concept and forceful reality structuring the ways in which biomedical production, consumption and innovation is grounded in the more or less coercive exploitation of a steadily expanding, largely informal labor force.

Conversely, the book provides a systematic and throughout comparative perspective on global clinical labor, thereby establishing knowledge beyond the single case study prevalent in social studies of science, technology, and biomedicine. Throughout, *Clinical Labor* examines jointly the technical-biological as well as the legal-contractual preconditions of biomedical innovation and bioeconomic expansion. For sure, novel biological and technological possibilities of intervention have provided the ground for the various developments described in the book; but far from engaging in a techno-centric explanation, Cooper and Waldby show that clinical labor does not only emerge from technological advances as such, but gain traction through various socio-economic and legal realignments, including a heavy deployment of “atavistic” principles of labor law. In this regard, *Clinical Labor* engages in a critical legal analysis that is closely tied to a critical analysis of bioethics. Analyzing the interrelationships between labor law and bioethics through the lenses of their shared implicit contractualism, Cooper and Waldby show, for instance, that “the principle of *informed consent* has historically evolved in a complex, often overlapping, yet largely undocumented dialogue with […] *contractual consent*” (p. 223, original emphasis). While the “historical mission” of bioethics was to protect biomedical subjects from harm and exploitation, bioethics has, perhaps unwittingly, helped to facilitate the consolidation of the informal clinical labor market and its globalization. Methodologically engaging in a veritably immanent critique, Cooper and Waldby’s analysis of bioethics goes well beyond common critical appraisals of bioethics that suggest, for instance, that bioethics was somehow “coopted” by the biomedical-industrial complex (see Turner 2009, for a discussion). Such a perspective provides powerful means to comprehensively understand and critically examine the role of bioethics within the overall deployment of the bioeconomy. Hence, Cooper and Waldby’s “clinical labor theory of value” does not so much resemble a self-enclosed theory (with capital T), but rather amounts to an intriguing theoretical perspective and generative framework of analysis. As such it invites to make the clinical labor perspective an integral part of the study of realignments of life sciences and political economy, and the study of contemporary biopolitics more generally.

In order to do so, we might suggest some further analytical dimensions that would complement and further substantiate the critical study of clinical labor relations beyond that what *Clinical Labor* has already accomplished.   In their analysis of the biopolitical reorganization of life, labor and value, only little attention is paid to questions of resistance and contestation at the level of clinical labor itself. Perhaps one could say that while Cooper and Waldby’s critique of clinical labor involves a thorough critique of (novel as well as sustained) forms of domination and exploitation, it falls short on the analysis of concrete power struggles. Here, further projects could benefit form, and in turn perfectly complement, studies of biopolitics that explore struggles and negotiations, as well as practices of subversion and contestation, at the micro- or meso-level of organization (see for instance Epstein 2007; Raman and Tutton 2010). Further, and closely related, there is the question of subjectivity. For sure, *Clinical Labor* excels in a detailed and differentiated analysis of the various subjects of clinical labor, exploring various subjects within the different subcategories of reproductive, experimental etc. labor. What Cooper and Waldby provide is a detailed map of subject positions, that is to say, structural positions coproduced within the global bioeconomy: subjects of and subjected by clinical labor. This type of analysis could be complemented with a more explicit focus on forms of subjectivation – that is to say, the concrete forms in which individuals and groups relate to and thereby “subjectify” clinical labor relations. Such an added double focus on contestation and subjectivation could shed new analytical light on pressing biopolitical questions. Take for instance cases where patients demand access to new experimental therapies, such as promising yet unproven stem cell treatments. By consuming such experimental treatments – many of them deployed “outside” the established norms of Western biomedicine and thus proscribed by bioethicists and regulators – these patients not only subvert bioethical norms, but also often participate in the clinical development of these therapies (Bharadwaj 2013). Here, clinical labor touches closely upon questions of biopolitical or “therapeutic citizenship” (Rose and Novas 2005; Nguyen 2005; Epstein 2007; Wehling 2010), a relationship that deserves further empirical as well as theoretical attention. It is also in these frontiers that biopolitical struggles are fought over a new emancipatory “ethics of the bio” (Bharadwaj 2013).

To conclude: Introducing "clinical labor” as an intriguing concept to explore hitherto marginalized aspects of biomedical innovation and neoliberal biopolitics, *Clinical Labor* is a welcome contribution to social studies of biomedicine. The book should concern everyone interested in the articulation of life, value and labor in global (bio-)economies. It provides detailed insights into the contemporary organization of the life sciences by exploring salient fields such as artificial reproductive technologies and clinical trials in global and truly intersectional perspective. In doing so, it implicitly suggests a framework for further analysis. Critical studies of biomedicine would benefit from further engaging with clinical labor, both empirically and conceptually.
